# Subjective cognitive decline in major depressive patients is associated with altered entropy and connectivity changes of temporal and insular region

**DOI:** 10.1038/s41398-025-03518-w

**Published:** 2025-09-01

**Authors:** Burak Yulug, Ali Yalcinkaya, Shair Shah Safa, Dila Sayman, Seyda Cankaya, Ayse Karakus, Ceyhun Sayman, Abdullah Burak Uygur, Emir Izzet Bircan, Aydogan Dogukan Ucak, Halil Aziz Velioglu, Lutfu Hanoglu, Adil Mardinoglu

**Affiliations:** 1 Department of Neurology and Neuroscience, Alanya Alaaddin Keykubat University, 07400 Antalya, Türkiye; 2 Functional Imaging and Cognitive-Affective Neuroscience Lab (fINCAN), Health Sciences and Technology Research Institute (SABITA), Istanbul Medipol University, 34815 Istanbul, Türkiye; 3 Department of Neurology, Istanbul Medipol University, 34815 Istanbul, Türkiye; 4 Department of Psychiatry, Alanya Alaaddin Keykubat University, 07400 Antalya, Türkiye; 5 Center for Psychiatric Neuroscience, Feinstein Institute for Medical Research, Manhasset, NY USA; 6 Science for Life Laboratory, KTH - Royal Institute of Technology, Stockholm, Sweden; 7 Centre for Host-Microbiome Interactions, Faculty of Dentistry, Oral and Craniofacial Sciences, King’s College London, London, UK

**Keywords:** Diseases, Neuroscience

## Abstract

Depressive cognitive impairment is seen in a significant number of patients with depression. However, it remains challenging to differentiate between patients with amnestic (those with subjective cognitive impairment complaints) and non-amnestic major depressive disorder, highlighting the urgent need for additional objective tools to help classify these patients more accurately. We analyzed cognitive state, alterations in regional entropy and functional connectivity measures of the brain between patients with major depression and healthy controls. The depressed cohort was categorized as either “amnestic” or “non-amnestic,” depending on self-reported experiences of forgetfulness. The superior temporal region and insula exhibited altered entropy and connectivity measures in individuals with depression and subjective cognitive impairment, which was correlated with impaired executive functions, a pattern not being evident in the control group. Our findings support the notion that insular and superior temporal entropic alterations are linked to subjective cognitive changes in the pathology of depression. These regions also hold potential as biomarkers for determining the underlying objective cognitive deficits in subjective cognitive complaints in patients with major depressive disorder (MDD). This underscores the need for improved diagnostic approaches and the implementation of practical dynamic neuroimaging modalities capable of addressing the current challenges in diagnosing subjective cognitive impairment in MDD, offering promise for the future management of patients with depression.

## Introduction

Cognitive impairment in depression is characterized by a range of cognitive deficits presenting with significant memory dysfunction, manifesting as difficulties in recalling past events, forming new memories, and maintaining attention [1]. These have a significant adverse impact on overall well‐being and individuals’ ability to perform daily tasks efficiently.

Several studies have indicated an increased risk of degenerative dementia in MDD patients with prominent amnestic symptoms [1–3]. For instance, Gao et al. suggested that depressive symptoms may act as a risk factor for subsequent cognitive decline, indicating the importance of accurately identifying patients with subjective cognitive impairment in order to apply preventive strategies against further cognitive decline as early as possible [4]. These findings suggest that depressive symptoms and subsequent dementia may be indicative of different manifestations of the same fundamental pathophysiological mechanisms. This in turn highlights the involvement of serotonergic degeneration and amyloid-beta build-up in the pathogenesis before the onset of cognitive impairment [1].

Fortunately, most patients with objective cognitive impairment in MDD are aware of their subjective cognitive impairments, however, identifying cognitive abnormalities in MDD is challenging even for experienced physicians due to the limited diagnostic power of current cognitive tools in patients with MDD due to the heterogeneity of both cognitive complaints related to MDD and the disease itself [5]. For instance, the severity of depression and anxiety, as well as patients’ medication and remission status, may bias objective evaluation of these so-called ‘emotionally driven’ subjective cognitive complaints by physicians. Gonda et al. classified cognitive dysfunctions in depression by their link to emotional symptoms, distinguishing between ‘hot’ cognitive functions, which are influenced by emotions, and ‘cold’ functions, which are not. Problems with ‘hot’ functions, often manifested as cognitive distortions where patients have a skewed perception of reality or situations, can be identified through patient interviews. In contrast, issues with ‘cold’ functions, which involve cognitive deficits mostly in executive functions, short term memory and attention in depressive patients, require neuropsychological or neurophysiological evaluations [6]. Although most patients are aware of their condition, some depressive patients with ‘hot’ cognitive dysfunctions may fail to recognize their cognitive deficits due to a distorted perception of reality or specific situations and may create a diagnostic difficulty for diagnostic tools.

Meanwhile, in addition to objective cognitive impairments, depressive patients may experience subjective cognitive concerns, which are believed to pose a potential risk for dementia [7]. For instance, Chin et al. evaluated one hundred and eighty patients with subjective cognitive complaints and found that the severity of subjective cognitive impairment was significantly correlated with patients’ depressive symptoms and self-focused attention scores, emphasizing the potential objective cognitive impact of subjective cognitive complaints [8]. Therefore, adopting a comprehensive diagnostic perspective by overcoming the limitations of these clinical evaluations might be crucial in identifying amnestic and non-amnestic MDD patients. To this end, novel neuroimaging techniques may prove beneficial in facilitating clinical diagnoses and tailoring antidepressant therapy options for different types of MDD patients [9].

The quantitative study of entropy evaluates brain complexity through computational methods, while reduced brain complexity is associated with various mental disorders. Successful implementations of brain entropy (BEN) analysis using functional magnetic resonance imaging data have yielded critical insights into disorders such as schizophrenia [10] and dementia [11]. For instance, a detrimental decline in BEN can be observed in schizophrenia, affecting the right middle prefrontal cortex, bilateral thalamus, right hippocampus, and bilateral caudate, while an increase in BEN is seen in the left lingual gyrus, left precuneus, right fusiform face area, and right superior occipital gyrus [12]. A variety of other mental disorders have also been associated with altered brain network entropy [13]. In particular, decreased BEN in superior frontal gyrus and increased BEN in parietal regions (angular gyrus, superior parietal lobule) and inferior temporal gyrus are reported in autism spectrum disorder [14]. In attention-deficit/hyperactivity disorder (ADHD), lower BEN in the sensorimotor network, default mode network, and visual network has been reported compared to healthy controls, with these changes significantly correlating with the severity of ADHD [15].

MDD, one such condition, is a significant mental disorder involving increased morbidity and mortality associated with reduced entropy in critical brain networks, and specific clinical characteristics [13]. For instance, the intensity of late-life depression is negatively associated with increased entropy in frontoparietal network activity, highlighting the importance of resting state temporal-spatial complexity in specific clinical aspects of depression. Similarly, several connectivity studies suggested the importance of altered connectivity in networks associated with affective and cognitive processing [13, 16] especially when it comes to temporal and insular regions [17, 18].

Our previous study showed that pulvinar connectivity plays a key role in discriminating between MDD patients with and without subjective memory impairment [19]. However, recent research suggests that in addition to subcortical connectivity alterations also the cortical entropy and connectivity changes of several cortical brain regions might be crucially important in the context of cognitive impairment in depression [20, 21].

Similar to diseased conditions, brain entropy (BEN) may also change in healthy individuals. For instance, a recent study evaluating 862 resting state fMRI of healthy individuals mentioned that lower resting-state BEN is associated with increased activation or deactivation in task-related brain regions [22]. An interesting study by Vivot et al. highlighted that meditation leads to an increase in entropy within the EEG, suggesting that these entropy changes may be a result of self-induced brain activity [23]. Wang et al. reported a significant negative correlation between years of education and brain entropy values in the default mode network (DMN) in healthy individuals [11]. Interestingly, the same paper also reports that in normal aging, BEN values increase and eventually plateau, whereas in Alzheimer’s disease, BEN values initially increase but then decrease instead of stabilizing, forming a reverse U-shape [11]. This model suggests that while a certain level of low BEN is essential for brain function, a detrimental BEN decline can lead to impaired cognitive performance, highlighting that although lower entropy may indicate reduced chaos in the system, the possibility of neural impairments should not be dismissed.

Additionally, there is a limited number of studies evaluating BEN alterations in individuals with subjective cognitive impairment (SCI). A recent study examining spatiotemporal entropy changes in EEG (epoc-based entropy) reported higher delta band entropy and reduced alpha and beta band entropy in Alzheimer’s disease patients compared to those with SCI [24]. Same study reported that patients with mild cognitive impairment (MCI) had significantly higher delta entropy values and lower alpha and beta entropy values than both AD and SCI patients [24]. Another study with similar results, showing higher theta band entropy in Alzheimer’s patients compared to SCI, suggested that EEG entropy could help clinicians diagnose Alzheimer’s disease with the accuracy of 91.6% (specificity = 100%, sensitivity = 87.8%) [25].

Our previous study showed that pulvinar connectivity plays a key role in discriminating between MDD patients with and without subjective memory impairment [19]. However, recent research suggests that in addition to subcortical connectivity alterations also the cortical entropy and connectivity changes of several cortical brain regions might be crucially important in the context of cognitive impairment in depression [20, 21]. Furthermore, previous studies highlighted the role of the insula and superior temporal region in degenerative cognitive disorders and cognitive awareness [26–28]. Based on this, we evaluated the cognitive state, alterations in regional entropy and functional connectivity measures of the brain between patients with major depression and healthy controls with a special focus on entropic and connectivity measures of STG and insula brain regions that might be valuable in revealing the underlying cognitive impairment in MDD. Despite solid clinical and imaging data, no studies to date have specifically examined entropic changes in specific cognitive regions and their relationship with connectivity changes and cognitive impairment in patients with depression presenting with subjective cognitive impairment.

## Material and methods

### Participants

In this cross-sectional study, clinicians at Alanya Alaaddin Keykubat University Hospital enrolled 31 outpatient depression patients with 15 amnestic and 16 non-amnestic subtypes, and 28 healthy controls between 18 and 64 years of age. Selection criteria for the patients included a diagnosis of MDD in accordance with the DSM-5 and by experienced psychiatrists. Tests of Depression: The severity of depression was measured by the HDRS. Inclusion/Exclusion Criteria: Excluding criteria for all subjects were the following: a) MMSE score less than 23, b) history of head trauma, stroke, and substance abuse/dependence currently or in the past, c) clinical evidence of any other significant current or past psychiatric or neurological illnesses, and d) usage of antidementia medication. Memory/Cognitive Measures Tested: These included the MMSE and ADAS-Cog. The study protocol was approved by the Ethics Committee of Istanbul Medipol University and written informed consent forms were obtained from participants after thoroughly informing them about the study protocol (Ethical Number: 10840098‐604.01.01‐E.19402)

### MRI data acquisition

Structural and resting‐state functional magnetic resonance imaging (fMRI) data were conducted using a Signa Explorer MR device (General Electric Company, USA) at Alanya Alaaddin Keykubat University, Turkey. Each individual’s T1‐weighted structural scans consisted of 190 slices (TR/TE: 8.1/3.7), FOV 256 × 256 × 190 mm (FHxAPxRL), and a voxel size of 1 × 1 × 1 mm. Eyes‐open resting state fMRI scan recordings were collected using an echo‐planar imaging sequence (EPI). The scanning process lasted approximately 12 min, and 300 volumes were recorded with the following parameters: TR 2230 ms, TE 30 ms, FOV 240 × 240 × 140 mm (RL × AP × FH), voxel size 3 × 3 × 4 mm, flip angle 770, and slice number 35. Before the scanning, all participants were instructed to keep their eyes closed, relax and move as little as possible, think nothing, and not fall asleep during the scanning.

### Entropy analysis of neuronal activity

We used dispersion entropy (DE) to analyse the complexity of signals. DE is a method based on Shannon entropy, as introduced by Azami et al. and Rostaghi and Azami [29]. It’s particularly efficient computationally and remains effective even when noise is present in the signal [30]. Compared to traditional multiscale techniques, DE offers a more dependable way to assess the underlying variability in neuronal activity over time and space [30].

The DE approach works by revealing dispersion patterns using the normal cumulative distribution function (NCDF). Each point in the signal sequence, $${x}_{i}=\{{x}_{1},\,{x}_{2},\,\ldots ,\,{x}_{N}$$} is assigned to a class. The dispersion pattern series ($${z}_{i})$$ is created by mapping each signal value to a class, ranging from 1 to $$c$$, according to the formula:

$${z}_{i}={round}({c}^{* }{y}_{i}+0.5)$$

Here *c*, represents the number of classes, and the NCDF maps the original signal values into a new sequence, ${y}_{i}=\left\{y1,y2,\ldots ,{y}_{N}\right\}$, with values between 0 and 1 [29]. In this method, the embedding dimension (mmm) defines the length of the sliding window used to group ${z}_{i}$ values into dispersion patterns, noted as $\left[{z}_{i},\ldots ,{z}_{i+m}\right].$ The time delay $(\tau$) represents the step length for the sliding window. Once dispersion patterns are identified, we calculate the probability of each pattern occurring, denoted as $p$, to determine the dispersion entropy using the formula:

$$EN=-\mathop{\sum }\limits_{i}^{{c}^{m}}{p}_{i{\ln}{p}_{i}}$$

For accurate DE calculation, the total number of possible dispersion patterns $({c}^{m})$ should be less than the signal length (*L*), as recommended by Rostaghi and Azami [29]. In our analyses, we fixed the parameters to $m=2,c=6$, $\tau$ = 1, ensuring ${c}^{m} < L$. We extracted time series data for each region of interest (ROI) using the Harvard-Oxford Cortical Atlas [31]. To maintain consistency across these parcelled time series, we standardized the data through z-scoring before calculating entropy. This approach, as noted by Gopal et al. and Koh et al. is especially effective for data sets where the mean and standard deviation remain stable over time [32]. After standardizing, we calculated the dispersion entropy (DE) for each identified ROI.

### Analysis of rsFC

Analyses of fMRI data were performed using CONN [33] (RRID:SCR_009550) release 21.a [34] and SPM [35] (RRID:SCR_007037) release 12.7771.

#### Preprocessing

Functional and anatomical data were preprocessed using a modular preprocessing pipeline [36] including realignment with correction of susceptibility distortion interactions, slice timing correction, outlier detection, direct segmentation and MNI-space normalization, and smoothing. Functional data were realigned using SPM realign & unwarp procedure [37] where all scans were coregistered to a reference image (first scan of the first session) using a least squares approach and a 6 parameter (rigid body) transformation [38] and resampled using b-spline interpolation to correct for motion and magnetic susceptibility interactions. Temporal misalignment between different slices of the functional data (acquired in ascending order) was corrected following SPM slice-timing correction (STC) procedure [39, 40], using sinc temporal interpolation to resample each slice BOLD time series to a common mid-acquisition time. Potential outlier scans were identified using ART [33] as acquisitions with framewise displacement above 0.9 mm or global BOLD signal changes above 5 standard deviations [41, 42], and a reference BOLD image was computed for each subject by averaging all scans excluding outliers. Functional and anatomical data were normalized into standard MNI space, segmented into grey matter, white matter, and CSF tissue classes, and resampled to 2 mm isotropic voxels following a direct normalization procedure [42] sing SPM unified segmentation and normalization algorithm [43, 44] with the default IXI-549 tissue probability map template. Last, functional data were smoothed using spatial convolution with a Gaussian kernel of 6 mm full width half maximum (FWHM).

#### Denoising

In addition, functional data were denoised using a standard denoising pipeline [36] including the regression of potential confounding effects characterized by white matter timeseries (5 CompCor noise components), CSF timeseries (5 CompCor noise components), motion parameters and their first order derivatives (12 factors) [38], outlier scans (below 20 factors) [41], session effects and their first order derivatives (2 factors), and linear trends (2 factors) within each functional run, followed by bandpass frequency filtering of the BOLD timeseries [45] between 0.008 Hz and 0.09 Hz. CompCor [46, 47] noise components within white matter and CSF were estimated by computing the average BOLD signal as well as the largest principal components orthogonal to the BOLD average, motion parameters, and outlier scans within each subject’s eroded segmentation masks. From the number of noise terms included in this denoising strategy, the effective degrees of freedom of the BOLD signal after denoising were estimated to range from 89.1 to 111.7 (average 104.9) across all subjects [42].

First-level analysis: Seed-based connectivity (SBC) maps were estimated to characterize functional connectivity patterns using ROIs from the Harvard-Oxford Cortical Atlas [31], identified based on entropy results, specifically in the superior temporal gyrus and insular cortex, within the CONN toolbox. Functional connectivity strength was represented by Fisher-transformed bivariate correlation coefficients from a weighted general linear model (weighted-GLM) [48]), defined separately for each pair of seed and target areas, modeling the association between their BOLD signal timeseries. In order to compensate for possible transient magnetization effects at the beginning of each run, individual scans were weighted by a step function convolved with an SPM canonical hemodynamic response function and rectified.

Group-level analyses were performed using a General Linear Model (GLM [48]). For each individual voxel a separate GLM was estimated, with first-level connectivity measures at this voxel as dependent variables (one independent sample per subject and one measurement per task or experimental condition, if applicable), and groups or other subject-level identifiers as independent variables. Voxel-level hypotheses were evaluated using multivariate parametric statistics with random-effects across subjects and sample covariance estimation across multiple measurements. Inferences were performed at the level of individual clusters (groups of contiguous voxels). Cluster-level inferences were based on parametric statistics from Gaussian Random Field theory [49, 50]. Results were thresholded using a combination of a cluster-forming p < 0.001 voxel-level threshold, and a familywise corrected p-FWE < 0.05 cluster-size threshold [51].

### Quality check

All participants’ MRI images were carefully reviewed by two researchers to verify their quality. It was confirmed that the anatomical and functional MRI images for all participants met the necessary standards for preprocessing. Following this, another round of assessment was performed. In this phase, the segmentation of T1-weighted anatomical images was checked, specifically focusing on the gray matter, white matter, and cerebrospinal fluid segmentations. The normalization of both functional and anatomical images to the MNI standard template was also visually inspected. For functional images, several parameters were evaluated, including the number of valid and invalid scans, maximum and average motion, as well as maximum and average global signal changes. Participants were excluded from the study if 25% or more of their scans were deemed invalid, or if they exhibited extreme values in maximum/average motion or maximum/average global signal changes. Extreme values were defined as those that exceeded Q3 + 3 IQR or fell below Q1 - 3 IQR, where Q1 and Q3 represent the first and third quartiles of the measure’s distribution, respectively, and the interquartile range (IQR) is the difference between Q3 and Q1 [52].

### Statistical analysis

Simple descriptive statistics and cognitive scores were analysed using Jamovi software (version 2.3.19.0). The Shapiro-Wilk test was used to check the normality of the variables. Continuous variables are presented as mean ± standard deviation (mean ± SD) or median (IQR) depending of the normality test of the variables and categorical variables as frequency (n) and percentage (%). The Independent T-Test and Mann-Whitney U tests were used to analyse ADAS score differences between the groups. A two-sided p-value ≤ 0.05 was interpreted as statistically significant. An effect size of 1.02 was calculated using G*Power.

Functional connectivity analyses were performed to identify group-level differences. A one-way ANCOVA was conducted to compare functional connectivity across the three groups (depression, amnestic depression, and control), while controlling for covariates including age, gender, education and HDRS scores. Following the ANCOVA, connectivity values were imported into the statistical software to perform post-hoc pairwise comparisons. Post-hoc analyses were conducted using Tukey’s HSD test to determine the sources of significant effects observed in the ANCOVA results. For variables violating the sphericity assumption, the Greenhouse-Geisser correction was applied to adjust degrees of freedom.

## Results

### Demographic features, clinical test scores

The participants’ demographic features and clinical test scores are summarized in Table 1. No significant differences were observed in terms of age (p = 0.065), but we observed significant differences in terms of education year (p = 0.002) and gender (p = 0.015) (Table 1). So, we included age, gender, and education year as covariates for our connectivity, entropy and correlation analysis. We observed significant differences in MoCA (p < 0.001), MMSE (p = 0.004), and ADAS scores (p < 0.001) between depression and control groups (Independent Student T test, Table 1). However, when we divided depression patients into amnestic and non-amnestic groups based on their own complaints of forgetfulness and analysed a total of three groups including the control group, significant differences were observed only in ADAS scores (between non-amnestic depression and control group p = 0.029, between amnestic depression and control group p = 0.009, Table 2) with no significant differences found in MOCA and MMSE tests. And we observed significant differences in digit span (p = 0.02*, Table 2) and sentence repetition (p = 0.012*, Table 2) between amnestic and non-amnestic depression groups which are subtest scores of MoCA. We also observed significant differences between amnestic depression and control groups in terms of digit span (p = 0.038, Table 2) and sentence repetition (p = 0.048*, Table2). Other sub-scores of MoCA test did not differ significantly between the three groups (p > 0.05) (Table 2).

**Table 1:** The participants’ demographic features.

	Depression Group (n = 31)	Control (n = 28)		
	Median (IQR)	Median (IQR)	p value	P Ancova
Age	40 (12.46)	33 (11.57)	0.065	
Education year	12 (4.43)	14 (3.76)	0.002*	
MOCA	24 (4.13)	27 (2.64)	0.002*	0.128
MMSE	28 (1.93)	29 (1.23)	0.008*	0.239
HDRS	10 (5.29)	2.5 (2.52)	<0.001*	<0.001*
Digit Span	1 (0.7)	2 (0.51)	0.017*	0.321
Sentence Repetition	1 (0.921)	2 (0.74)	0.022*	0.486
Gender (female n, (%))	24 (%77)	13 (%46)	0.015*	

There were no significant differences in Mann-Whitney U Test in terms of age, education year and gender. Digit Span, a subscore of MOCA differed between the Depression and control groups.

*Indicates a statistically significant *p*-value

**Table 2:** Significant differences were observed in ADAS, HDRS, sentence repetition, and Digit Span between groups.

Tests	Groups			Mean Difference	P tukey
MoCA	Non-Amnestic D	-	Amnestic D	0.45	0.87
		-	Control	−0.92	0.49
	Amnestic D	-	Control	−1.37	0.29
MMSE	Non-Amnestic D	-	Amnestic D	1.04	0.12
		-	Control	−0.08	0.98
	Amnestik D	-	Control	−1.12	0.08
HDRS	Non-Amnestic D	-	Amnestic D	−1.57	0.6
		-	Control	7.3	< 0.001*
	Amnestic D	-	Control	8.87	< 0.001*
Digit Span	Non-Amnestic D	-	Amnestic D	0.58	0.02*
		-	Control	0.05	0.95
	Amnestic D	-	Control	−0.52	0.038*
ADAS	Non-Amnestic D	-	Amnestic D	−0.55	0.74
		-	Control	1.75	0.029*
	Amnestic D	-	Control	2.31	0.009*
Sentence Repetition	Non-Amnestic D	-	Amnestic D	0.75	0.012*
		-	Control	0.15	0.77
	Amnestik D	-	Control	−0.6	0.048*

There is no significant difference in MoCA and MMSE.

### Entropy results

#### Between-group differences in the entropy of the brain regions

**Fig. 1:**
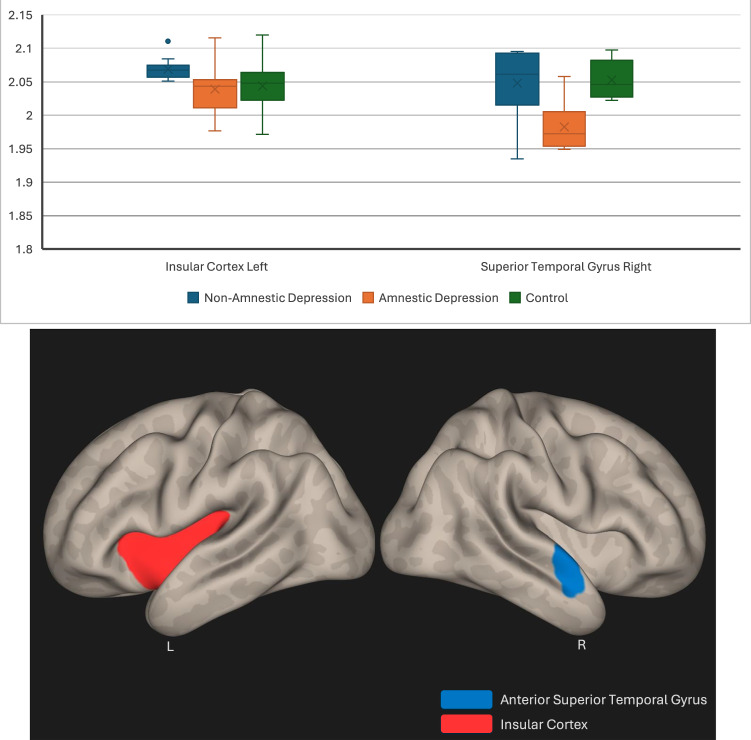
The left insular cortex and right anterior superior temporal gyrus demonstrated decreased entropy in amnestic depressive patients compared to non-amnestic depression patients.

The left insular cortex and right anterior superior temporal gyrus demonstrated decreased entropy in amnestic depressive patients compared to non-amnestic depression patients (p < 0.05) (Fig. 1).

### Connectivity analysis

**Fig. 2:**
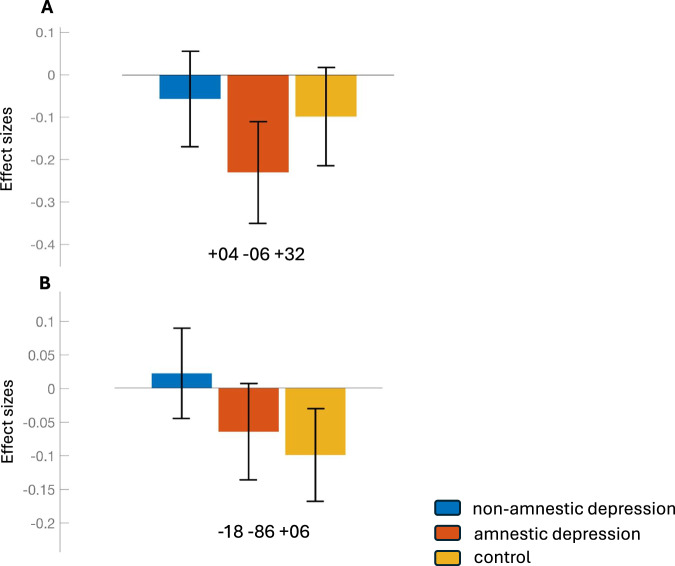
Effect size of ROIs showing functional connectivity differences between non-amnestic depression, amnestic depression, and control groups. The effect sizes (Fisher z-transformed correlation coefficients) are shown for three groups: the blue bars represent the non-amnestic depression group, the red bars represent the amnestic depression group and the yellow bars represent the control group. The effect size reflects the strength of functional connectivity in ROIs, which shows significant connectivity differences between the three groups. Panels **A** and **B** represent the left posterior STG, and right insular cortex seeds, respectively. The coordinates on the x-axis correspond to the peak MNI coordinates (from Table 3), related to the ROIs that exhibited functional connectivity differences between the three groups.

**Fig. 3:**
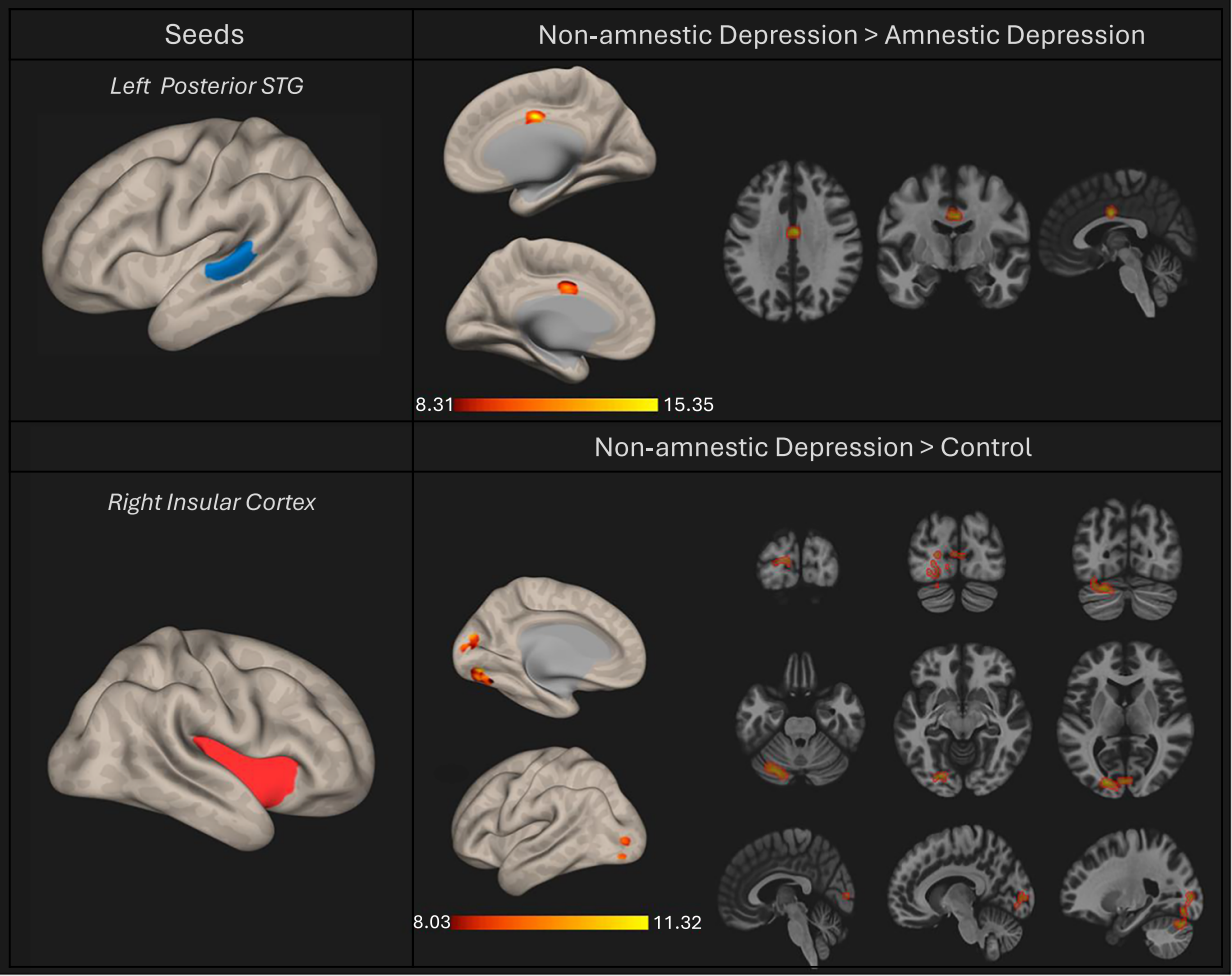
The seed-based connectivity results of the left posterior STG and right insular cortex. The left posterior STG, in the non-amnestic depression group, exhibited higher connectivity to the anterior cingulate compared to the amnestic depression group. The right insular cortex, in the non-amnestic depression group exhibited higher connectivity to the left cerebellum and bilateral occipital regions compared to the control group. The red patches indicate an increase in functional connectivity. The colour bars present the F-statistics. The regions depicted on brain maps are described in Table 3. Abbreviation: STG, superior temporal gyrus.

We observed decreased functional connectivity between the left Posterior Superior Temporal Gyrus and Anterior Cingulate cortex in the Amnestic Depression group compared to the Non-amnestic Depression and Healthy control group. (Table 3) (Figs. 2 and 3).

**Table 3:** The left posterior temporal gyrus showed decreased connectivity in the amnestic depression group compared to the non-amnestic depression group.

Seeds	Cluster Location	MNI coordinates	size	F	*p*-FWE
**Left Posterior Superior Temporal Gyrus**	Anterior Cingulate	+04 −06 + 32	106	17.89	0.0033
**Right Insular Cortex**	Left Cerebellum Crus1	−18 −86 + 06	488	32.53	0.0000
	Left Occipital Fusiform Gyrus				
	Left Cerebellum 6				
	Left Intracalcarine Cortex				
	Left Occipital Pole				
	Right Intracalcarine Cortex				

The right insular cortex showed increased connectivity in the non-amnestic depression group compared to the control group. The p-value assesses the difference between the three groups using one-way analyses of covariance.

We used age, education year, gender, and HDRS scores as covariates in all connectivity analyses. We also found that the right insular cortex in the non-amnestic depression group showed higher connectivity to the left cerebellum, left occipital fusiform gyrus, left occipital pole, and the bilateral intracalcarine cortex compared to the control group. (p-Tukey < 0.05) (Table 3) (Figs. 2 and 3).

### Correlation analysis

Partial correlation analysis, controlling for age, gender, education year and HDRS score showed a significant positive correlation between digit span and right superior temporal region (r = 0.457*, p = 0.017) and left insular cortex brain entropy (BEN) alteration (r = 0.397*, p = 0.04) which was evident in depression group with amnestic and non-amnestic patients (Supplementary Table 1). We also observed a positive correlation between sentence repetition scores, a subtest of MoCA, and functional connectivity between left Superior Temporal Gyrus and Anterior Cingulate cortex (Supplementary Table 2). Furthermore, a significant correlation between the connectivity and entropic alteration was observed in depressive patients which was not evident in the control group (Pearson’s r: 0.557, p: 0.002). We used age, education year, gender and HDRS scores as covariates in all connectivity and correlation analyses. Additionally, there is not any significant correlation between HDRS scores and brain entropy alterations of right superior temporal gyrus and left insular cortex (p > 0.05) (Supplementary Table 3).

## Discussion

In the present study, we showed that patients with MDD with subjective cognitive impairment exhibited different regional brain entropy and connectivity values of STG and insula to those of individuals without subjective memory complaints, a phenomenon that was particularly evident in specific cognitive subdomains, such as digit span and sentence repetition. We also observed a significant positive correlation between digit span and brain entropy (BEN) alterations in the right superior temporal region and left insular cortex. Additionally, a positive correlation was found between sentence repetition scores and functional connectivity between the left superior temporal gyrus and anterior cingulate cortex.

This suggests that subjective awareness of memory deficits reflects an underlying objective executive dysfunction associated with the entropic and connectivity alterations of these brain regions which are responsible for cognitive functions.

The superior temporal gyrus (STG) and insula are two key areas implicated in this complex interaction. For instance, STG is involved in both memory and self-awareness processes which may impair individuals’ ability to reflect on their own cognitive and emotional states. A good example of this is a recent study by Liu et al., who observed that damage to STG correlates with deficits in understanding, thus emphasizing its importance to cognitive functioning [53]. Similarly, Wu et al. showed altered regional homogeneity in the STG in patients with mild cognitive impairment (MCI) [54]. Task-based functional MRI studies have further confirmed the role of STG in cognitive tasks. For instance, Ji et al. reported hypoactivation of the STG in patients with Parkinson’s disease with MCI during cognitive tasks, suggesting that reduced activity in this region is associated with cognitive deficits [55].

The role of the STG in executive functions was suggested in a recent study by Yue et al, who identified it as a critical node in the default mode network, a crucial network regulating cognitive functions [56]. This was consistent with a subsequent study showing a positive correlation between right STG gray matter volume and overall cognitive performance in patients with vascular cognitive impairment [57]. Supporting our results of impaired specific cognitive functions, several other reports concerning depression have indicated the role of the superior temporal region in the processing of language, mood and execution which are essential to understanding emotional states and self-awareness in depressive disorders [58].

Furthermore, neuroimaging studies have consistently demonstrated that STG, particularly in the left hemisphere, plays a critical role in the processing of sentence comprehension [59] and repetition [60], explaining the fact why damage to the STG, is associated with significant impairments in sentence repetition and executive functions in stroke patients [60]. These findings align well with recent literature showing that reduced activity and connectivity within STG are associated with impaired sentence repetition and executive performance in individuals with depression [61, 62]. Considering all this evidence, the realization that individuals with MDD exhibit reduced right STG gray matter volume potentially responsible for diminished capacity to perceive one’s own mental health condition and recognize one’s own depressive symptoms was therefore unsurprising [63–65].

Confirming the entropic difference between amnestic and non-amnestic groups in our study, the insula also may have a particular role in executive functions and cognitive awareness due to its capacity to integrate information from various brain regions, such as the amygdala and other limbic structures [66, 67] This aligns with our findings of altered insular connectivity results in non-amnestic patients compared to controls which was not evident in amnestic patients. Recent data suggesting that the insula is integral to the salience network, which is crucially important for detecting and integrating salient stimuli from the environment, thereby influencing cognitive processes such as attention, decision-making, and emotional regulation [68, 69]. Additional evidence of the role of the insula in cognition comes from studies identifying the anterior insular cortex as crucially important for interoceptive awareness and attention, linking physiological states to cognitive and emotional experiences [68, 70]. More specifically, the insula is intimately associated with executive functions that may influence the manifestation of cognitive impairment. From the neurobiological perspective, alterations in synaptic transmission within the insula have been also linked to impaired cognition through the involvement of long-term depression [71]. This is consistent with previous studies showing that dysfunction in this area leads to impaired decision-making abilities, particularly in conditions such as dementia, in which interoceptive processing is compromised [72]. The role of the insula in integrating emotional and cognitive information has also been confirmed in psychiatric conditions characterized by cognitive impairment, such as mood and psychotic disorders [73, 74]. A good example of this is that individuals at clinically high risk for psychosis exhibit anatomical variations in the insula that relate to cognitive deficits [75].

Similarly to both neurodegenerative and healthy conditions, the role of insula in executive functions has additionally been confirmed in MDD. Specific dysfunction in this area may lead to critical deficits in the ability of an individual with MDD to maintain cognitive functions, thus further contributing to increased morbidity in depression, as previously shown for several neurodegenerative conditions. This is consistent with therapeutic neuromodulation studies suggesting that modulating the anterior and posterior insular regions restores some degree of awareness and executive functions in healthy individuals and patients with early psychosis, thus highlighting its potential for cognitive enhancement [76–78].

Our findings of altered insular and superior temporal gyrus entropy and connectivity values in subjective cognitive impairment in MDD suggest that the decreased brain entropy pattern along with appropriate connectivity changes in these relevant regions, may accurately reflect an underlying objective deficit prominent in specific cognitive sub-domains that was undetectable through conventional cognitive tests. Furthermore, our findings, along with those of previous studies of dementia support the idea that decreased entropy and increased connectivity in these regions are directly correlated with increased cognitive decline, a finding that has been also confirmed both for the insular and superior temporal regions in recent studies [79, 80]. Additionally, the significant correlation between connectivity and entopic alterations in STG further implies the role of STG in subjective cognitive impairment in MDD.

Additionally, we observed greater connectivity between the right insular cortex and the left cerebellum, left occipital fusiform gyrus, left occipital pole, and bilateral intracalcarine cortex in the non-amnestic depression group compared to the control group. This finding suggests that these regions may play a role in the pathophysiology of depression due to their close relationship with limbic networks, which are essential for emotion regulation [81, 82]. For instance, Marwood et al. reported decreased activation in the insular and anterior cingulate cortices in patients with depression and anxiety following psychotherapy [82]. Similarly, altered cerebellar connectivity has been observed in mood disorders, as well as in patients with depression, particularly in drug-resistant cases [83]. Xu et al. investigated brain entropy changes in MDD patients after electroconvulsive therapy, a widely used method known for its antidepressant effects, and observed a significant reduction in BEN in the posterior cerebellar lobule, highlighting the cerebellum’s potential involvement in mood regulation [84]. On the other hand, decreased BEN in medial orbitofrontal cortex has been observed in MDD patients [85, 86]. The same study also reported that after 8 weeks of antidepressant therapy, greater symptom improvement was associated with higher initial BEN in the medial orbitofrontal cortex and hippocampus, along with a significantly larger BEN reduction in the medial orbitofrontal cortex, but a smaller decrease in the motor cortex, temporal cortex, fusiform gyrus, and visual cortex [85].

From the clinical perspective, our results may suggest earlier literature that executive and language function failures are more detectable than those in other cognitive domains in daily routines. This phenomenon appears to be a distinct process from the depressive state and may present as subjective cognitive impairment as an earlier sign and predictor of subsequent memory decline [87–89].

For example, Wang et al. noted that cognitive assessments performed without the inclusion of executive function tests will miss a significant percentage of patients with clinically relevant cognitive impairments [90]. This was also reported by Schofield et al., who showed that subjective memory complaints are not consistent with objective cognitive assessments, at least as far as executive functions are concerned [91]. These findings also have implications for clinical practice, whereby most of these assessments merely designed to study memory may underestimate vital executive dysfunctions, which might serve as a guide to treatments and management. Hence, our findings and the recent literature suggest that the assessment of executive and language functions should be holistic, much like the assessment of memory, to conceptualize and thus intervene appropriately in cognitive impairments within the clinical setting of depressive subjective cognitive impairment.

Despite providing valuable data regarding entropy and connectivity alterations in depressive cognitive impairment, there are several limitations to this study requiring discussion. Since the amnestic depression group did not differ from other depressed patients in terms of the type and duration of antidepressant therapy or HDRS severity, this group may over-represent those individuals, a group that may require special evaluation in further studies. However, after adjusting the HDRS scores across all groups, including even minor HDRS scores in the control group, differences in cognitive scores between the amnestic and non-amnestic depression groups persisted.

Moreover, our depressive group was not drug-naive, and the treatment may have influenced cognitive factors and brain connectivity over time, although the types of drugs and time of treatment were similarly distributed between the groups. Furthermore, there is no valid method for calculating self-reported cognitive awareness in depression, which complicates statistical comparisons between self-reported scores and functional connectivity. However, beyond merely replicating general observations of altered regions in depression, our findings extend our understanding of cognition by linking subjective cognitive awareness with specifically altered entropy and connectivity measures in MDD patients.

In conclusion, combined with other research, our findings support the notion that insular and superior temporal entropic alterations make a specific contribution to subjective cognitive changes in the pathology of depression. They also highlight the potential use of these regions as a biomarker for determining the underlying objective cognitive deficits in subjective cognitive complaints in patients with MDD. It therefore underscores the need for the improvement of diagnostic approaches toward the implementation of practical dynamic neuroimaging modalities capable of addressing the present challenges in diagnosing subjective cognitive impairment in MDD, and that holds out promise for future management of depressive patients.

## Supplementary information

Supplemental Tables

## Data Availability

The datasets used and/or analysed during the current study are available from the corresponding author on reasonable request due to ethical reasons.
