# Supplementary material for: Subjective cognitive decline in major depressive patients is associated with altered entropy and connectivity changes of temporal and insular region
Source: Transl Psychiatry. 2025 Sep 1;15:335. doi: 10.1038/s41398-025-03518-w (PMC12402481; doi:10.1038/s41398-025-03518-w)
Supplement: Supplementary file 1 — Supplemental Tables [file 41398_2025_3518_MOESM1_ESM.docx]

**Supplementary Table1**: Partial Correlation between Digit span scores and brain entropy alterations of right Superior Temporal gyrus and left Insular Cortex:

|  | | | | | | | | | | | | |  |
| --- | --- | --- | --- | --- | --- | --- | --- | --- | --- | --- | --- | --- | --- |
|  |  | | | **Digit Span** | | **Entropy of right STG** | | | **Entropy of left IC** | | | |  |
| Digit Span |  | Pearson's r |  | | — | |  |  | |  |  |  | |
|  |  | p-value |  | | — | |  |  | |  |  |  | |
| Entropy of right STG |  | Pearson's r |  | | **0.457*** | |  | — | |  |  |  | |
|  |  | p-value |  | | **0.017** | |  | — | |  |  |  | |
| Entropy of Left IC |  | Pearson's r |  | | **0.397*** | |  | 0.474 | |  | — |  | |
|  |  | p-value |  | | **0.040** | |  | 0.013 | |  | — |  | |
| Note. controlling for gender, education year, age, and HDRS scores. | | | | | | | | | | | | |  |
| Note. * p < .05 | | | | | | | | | | | | |  |
|  | | | | | | | | | | | | |  |

*The table shows the partial correlation analysis between digit span scores and the brain entropy alterations of superior temporal gyrus and left insular cortex. Age, gender, education year and HDRS score are adjusted. As the result the analysis showed a significant positive correlation between digit span scores and brain entropy alterations of right superior temporal region (r=0.457*, p=0.017) and left insular cortex (r=0.397*, p=0.04) in whole depression group (amnestic + non-amnestic). The correlation mentioned is not present in control group. (Abbreviations: STG: Superior Temporal Gyrus; IC: Insular Cortex.)

**Supplementary Table 2:**Partial Correlation between sentence repetition scores and functional connectivity of Superior Temporal Gyrus with Anterior Cingulate Cortex.

|  | | | |
| --- | --- | --- | --- |
|  |  | **FC between left STG and ACG** | **Sentence Repetition** |
| **FC between left STG and ACG** | **Pearson's r** | — |  |
|  | **p-value** | — |  |
| **Sentence Repetition** | **Pearson's r** | **0.29*** | — |
|  | **p-value** | **0.029** | — |
| Note. Controlling for gender, age, education year, and HDRS scores. | | | |
| Note. * p < .05  *Table shows a significant positive correlation between sentence repetition scores, a subtest of MoCA and the functional connectivity between left Superior Temporal Gyrus and Anterior Cingulate Cortex (r=0.29, p=0.029). Age, gender, education year and HDRS scores are adjusted.  (Abbreviations: FC: Functional Connectivity; STG: Superior Temporal Gyrus; ACG: Anterior Cingulate Cortex.) | | | |

**Supplementary Table 3:** Partial Correlation between HDRS scores and brain entropy alterations of right Superior Temporal gyrus and left Insular Cortex.

|  | | | | | | | | | |
| --- | --- | --- | --- | --- | --- | --- | --- | --- | --- |
|  | |  | | **Entropy of left IC** | | **Entropy of right STG** | | **HDRS** | |
| **Entropy of left IC** |  | Pearson's r |  | — |  |  |  |  |  |
|  |  | p-value |  | — |  |  |  |  |  |
| **Entropy of right STG** |  | Pearson's r |  | 0.465 |  | — |  |  |  |
|  |  | p-value |  | 0.013 |  | — |  |  |  |
| **HDRS** |  | Pearson's r |  | -0.145 |  | 0.025 |  | — |  |
|  |  | p-value |  | **0.461** |  | **0.901** |  | — |  |
| Note. Controlling for gender, education year, and age.  *The table shows that there is not any significant correlation between HDRS scores and brain entropy alterations of right Superior Temporal gyrus and left Insular Cortex (p>0.05). Age, gender and years of education scores are adjusted.  (Abbreviations: IC: Insular Cortex; STG: Superior Temporal Gyrus.) | | | | | | | | | |
|  | | | | | | | | | |
|  | | | | | | | | | |
